# Effects of Alkalinity and pH on Survival, Growth, and Enzyme Activities in Juveniles of the Razor Clam, *Sinonovacula constricta*

**DOI:** 10.3389/fphys.2018.00552

**Published:** 2018-05-18

**Authors:** Peng Maoxiao, Ye Bo, Liu Xiaojun, Niu Donghong, Lan Tianyi, Dong Zhiguo, Li Jiale

**Affiliations:** ^1^Key Laboratory of Exploration and Utilization of Aquatic Genetic Resources, College of Fisheries and Life Science, Shanghai Ocean University, Shanghai, China; ^2^Co-Innovation Center of Jiangsu Marine Bio-industry Technology, Huaihai Institute of Technology, Lianyungang, China

**Keywords:** carbonate alkalinity, enzyme activity, growth, inland saline water, pH, *Sinonovacula constricta*

## Abstract

In order to clarify the possibility of rearing razor clams (*Sinonovacula constricta*) in inland saline water (ISW) and to facilitate their breeding under these stressful conditions, we performed semi-static acute and chronic toxicity tests to determine the effects of carbonate alkalinity (CA) and pH on the survival and growth rate, and critical metabolic enzyme activity in juvenile of *S. constricta* (JSC). (1) Acute toxicity test. As the water *C*_A_ increased from 1.22 to 45.00 mmol L^-1^, the survival rate decreased significantly, which was exacerbated by the increase in the pH. When the water *C*_A_ was set at 2.5 mmol L^-1^, the 48 h lethal concentration 50% (LC_50_) for JSCs with respect to pH was 9.86. When the water pH was 9.0, 9.5, and 10.0, the 48 h LC_50_ values for JSCs with respect to *C*_A_ were 10.38, 8.79, and 3.11 mmol L^-1^, respectively. (2) Chronic toxicity test. Four experimental groups comprising the control, CAS, pHS, and CA-pHS were designated according to the target ISW data. After 3 months of stress, the JSC survival rate in each group exceeded 85%, but survival was significantly lower in the CA-pHS group than the control group (*p* < 0.05) in the first month. For the JSCs in various groups, the shell length growth rate (SGR) and weight gain (WG) rate were significantly lower in the CA-pHS group than the other groups (*p* < 0.05 for SGR; *p* < 0.001 for WG) in the first month. However, the difference in the growth rate among groups decreased in the next 2 months. For the JSCs in the CA-pHS group, the oxygen consumption, ammonia-N excretion, Na^+^/K^+^-ATPase, aspartate aminotransferase, and superoxide dismutase levels were significantly higher than those in the other groups during the first month, but there were no significant differences between the groups subsequently. The acetylcholinesterase and lysozyme levels did not differ significantly among groups during stress for 3 months. The integrated biomarker response index showed that stressors comprising high pH and *C*_A_ could be tolerated well by JSCs over long periods of stress. These results indicate that water *C*_A_ and pH together affect the survival, growth, and physiological activity of JSCs. *S. constricta* is suitable for culture in ISW.

## Introduction

Throughout the world, saline-alkali soils provide low yields (Qadir et al., 2001; Wang et al., 2011). The development and utilization of saline-alkali soil and water resources is a focus of a research. In the 1920s, the former Soviet Union began breeding experiments with fish, shrimp, and shellfish in inland saline water (ISW), but with very little success (Yao et al., 2010). In Israel, inland saline aquaculture, known as “desert aquaculture” began operating commercially in the late 1980s and it is characterized by the raising of finfish in brackish geothermal water from deep aquifers discovered in the 1940s (Allan et al., 2009). In Pakistan and Iran, marine fish and freshwater fish have been considered suitable for cultivation in brackish water, showing a high degree of adaptability to changing water environments (Salati et al., 2014; Rahim et al., 2017; Malik et al., 2018). In New Zealand, marine fish (Fielder et al., 2001; Doupé et al., 2005; Ingram et al., 2015) and crustaceans (Prangnell and Fotedar, 2005; Tantulo and Fotedar, 2006) have been farmed in ISW after selecting the appropriate water quality or improving the water quality (regulate the main ionic components of ISW). This method is also used in Australia (Doroudi et al., 2006) and the United States (Boyd et al., 2007). ISW aquaculture is now practiced worldwide, where it was inspired by the success of *Litopenaeus vannamei* aquaculture in Thailand using a brine solution (Roy et al., 2010), which expanded worldwide subsequently, including in Brazil, China, Ecuador, Mexico, Thailand, the United States, and Vietnam (Dinh, 2015). However, the cultivation of shellfish in ISW has been only reported sporadically, such as with *Mytilus edulis* (Dinh and Fotedar, 2016), *Haliotis laevigata* (Doupé et al., 2003), *Trochus niloticus* (Lee, 1997), *Crassostrea gigas*, and *Saccostrea glomerata* (Ingram et al., 2015).

In China, the saline-alkali land areas comprise 9970 hm^2^ with 3067 hm^2^ of inland saline-alkali waters (He et al., 2010), which are characterized by high carbonate alkalinity (*C*_A_) and pH, imbalanced ions, and water with a complex chemical composition (Wang et al., 1997; Shentu et al., 2000). Some saline-alkali waters have been used for culture and aquaculture, but most of them have been in a barren state for a long time (Lv et al., 2012). Freshwater fish (Zhang et al., 1999) and seawater shrimp (Fang et al., 1995; Hu et al., 2000; Liu et al., 2008) are cultured the most widely in China’s ISW fisheries. Few studies have investigated the breeding of shellfish in Chinese ISWs, where only one study determined the toxic effects of several saline-alkali factors on *Cyclina sinensis* (Lin et al., 2012). Utilizing ISW as a water resource for aquaculture in China will help to improve the agricultural ecological environment, adjust the agricultural economic structure, and increase the outputs of farmers.

*Sinonovacula constricta* (Chinese razor clam) is the fourth most important commercial marine bivalve species and it is widely distributed in the intertidal zones and estuarine areas of the western Pacific Ocean (Xie et al., 2015). *S. constricta* can adapt to a wide range of temperature and salinity conditions (Lin and Wu, 1984), and it is characterized by a short production cycle, low production costs, and high production efficiency (Wu and Xv, 2000; Niu et al., 2007). Aquaculture research into *S. constricta* has focused on growth (Niu et al., 2015), development (Niu et al., 2016), and disease control (Feng et al., 2012; Peng et al., 2016). Several studies have focused on water quality factors such as salinity (Lin and Wu, 1990), metal ions (Wu et al., 2003), inorganic pollutants (Chen et al., 1984), organic pollutants (Jiang et al., 2015), and pH (He et al., 2017), whereas none have considered the effects of *C*_A_ and pH on the survival and growth of *S. constricta*. In addition, shellfish can fix calcium carbonate and dietary algae in the water to achieve an ecological balance in aquaculture (Shumway et al., 2003; Tolley et al., 2004; Dumbauld et al., 2009; Guo et al., 2017). Therefore, in this study, we investigated the effects of *C*_A_ and pH (high CO_3_^2-^, HCO_3_^-^, and OH^-^) on the survival, growth, and physiological characteristics of juvenile *S. constricta* (JSC), and aims to confirm whether *S. constricta* is suitable for use as a breeding species in the inland saline-alkali waters of China.

## Materials and Methods

Animals were handled according to the guidelines for the care and use of animals for scientific purposes set by the Institutional Animal Care and Use Committee of Shanghai Ocean University, Shanghai, China. The test animals were non-endangered animals and were artificially propagated larvae.

Healthy JSCs were obtained from Donghang Farm, Sanmen City, Zhejiang Province, China. In the experiments, 100 JSCs were randomly selected to measure their weight and length. The average body weight and average shell length of the JSCs were 0.0164 ± 0.0027 g and 0.6184 ± 0.069 cm, respectively. Freshwater mixed with artificial sea salts (Red Sea, Red Sea Fish Pharm Ltd., Israel) was used to prepare different salt concentrations in artificial seawater (ASW) for the experiments. In this study, setting the salinity in 6 ppt. It is based on the meteorological and ISW water quality data in the Jingtai region of Gansu Province, northwestern China, which the salinity of most carbonate water is around 6 ppt (Dou, 2006; Lai et al., 2007; Wang and Shi, 2010; Yao et al., 2010). The water temperature detected in the experiments was 20–22°C. In our experiments, in each group of tests, the *C*_A_ in the water was adjusted using Na_2_CO_3_ and NaHCO_3_, and the pH was adjusted with 0.1 mol L^-1^ HCl and 0.1 mol L^-1^ NaOH. The *C*_A_ was detected by acid-base titration with phenolphthalein and methyl orange-aniline blue as indicators (Xiao, 2004). Three separate sets of experiments were performed for every group.

### Acute Toxicity Test

#### Test Methods

During the test period, the pH and *C*_A_ values of the test water varied due to the CO_2_ in the air and the physiological activities of the JSCs. Therefore, the values were stabilized by changing 100% of the water each day. Petri dishes (9 cm in diameter) and 80 mL of the test water were used for each group, where 6 ppt ASW was set as the control group (Lai et al., 2007; Yao et al., 2010). The JSC survival rate (SR) after 48 h was the test result.

#### JSCs With Different pH Values Under the Same *C*_A_

The normal seawater *C*_A_ ranges between 2 and 3 mmol L^-1^, so *C*_A_ was set at 2.5 mmol L^-1^ (measured as 3.74 ± 0.12 mmol L^-1^) in this test. The pH groups were set at 7.5, 8.0, 8.5, 9.0, 9.5, 10.0, and 10.5. The control group was set at 6 ppt ASW, without additional adjusting *C*_A_, and the pH was 8.20. Three separate groups (each containing 30 JSCs) were tested at each pH groups.

#### JSC With Different *C*_A_ Values at the Same pH

The pH of natural carbonate alkaline water is generally high (Shi, 2009), so in this test, we set the pH at 9.0, 9.5, and 10.0. In preliminary experiments, lower survival occurred at pH 9.0 and *C*_A_ 20 mm L^-1^, so the design of each group of experiments is shown in **Table 1**. Three separate sets of groups (each containing 30 JSCs) were tested.

**Table 1:** Designed and actual carbonate alkalinity (*C*_A_) levels with different pH values.

pH 9.0		pH 9.5		pH 10.0	
Setting *C*_A_ (mmol⋅L^-1^)	Measured *C*_A_ (mmol⋅L^-1^)	Setting *C*_A_ (mmol⋅L^-1^)	Measured *C*_A_ (mmol⋅L^-1^)	Setting *C*_A_ (mmol⋅L^-1^)	Measured *C*_A_ (mmol⋅L^-1^)
0	1.22 ± 0.08	0	1.38 ± 0.04	0	1.84 ± 0.25
5	4.57 ± 0.33	5	4.13 ± 0.06	1	2.97 ± 0.20
15	14.47 ± 0.42	10	10.66 ± 0.57	3	4.16 ± 0.11
20	19.70 ± 0.59	15	15.63 ± 0.58	5	4.74 ± 0.32
30	30.20 ± 0.50	20	18.93 ± 0.90	7	6.77 ± 0.16
40	38.00 ± 0.85	30	26.28 ± 1.48	10	9.41 ± 0.34
50	45.00 ± 0.93	40	38.51 ± 0.54	13	11.96 ± 0.11
–	-	50	44.58 ± 1.82	15	14.10 ± 0.21
–	-	–	-	20	17.94 ± 0.41

### Long-Term Toxicity Test

Beach mud was used in the long-term toxicity test. The beach mud was collected from East China Sea, Lingang new city, Shanghai city, China. The collected mud was treated for use in the test, as follows. First, a 2.00-mm grade sieve was used to remove impurities from the mud. Second, the mud was resuspended three times in fresh water (mud:fresh water = 10:40 L); Finally, the mud was dried at 80°C and then dissolved in the test water. After preliminary acclimation for 10 days in ASW (10 ppt), 1200 JSCs were selected randomly and transferred into a tank (40 cm × 40 cm × 65 cm). Each tank contained 10.4 L of test mud, 12 L of test water, and 100 JSCs. Four groups were designed to test the control, pH stress (pHS), *C*_A_ stress (CAS), and pH plus *C*_A_ stress (CA-pHS) conditions. In each group, the JSCs were acclimated gradually over 8 days (**Table 2**). The final stress conditions comprised: control (6 ppt salinity, 1.22 ± 0.08 mmol L^-1^ *C*_A_, 8.23 ± 0.04 pH), pHS (6 ppt salinity, 1.21 ± 0.18 mmol L^-1^ *C*_A_, 10.00 ± 0.03 pH), CAS (6 ppt salinity, 8.34 ± 0.36 mmol L^-1^ *C*_A_, 8.29 ± 0.07 pH), and CA-pHS (6 ppt salinity, 4.98 ± 0.28 mmol L^-1^ *C*_A_, 9.20 ± 0.02 pH). During the test period, 100% of the water was changed each day to avoid CO_2_ in the air affecting the pH value and *C*_A_. The JSCs were fed once each day (16:00) with *Chaetoceros calcitrans*, where each tank containing 12 L of test water had a concentration of 200–240 *C. calcitrans* cells μL^-1^ (Zhu et al., 2010). Before feeding, the *C. calcitrans* culture solution was subjected to centrifugation (1000 g min^-1^ for 10 min) and resuspended twice in the test water. Throughout the stress test period of 100 days, each group of JSCs was analyzed to determine the per month survival rate (PMSR), weight gain (WG) rate, shell length growth rate (SGR), oxygen consumption rate, ammonia excretory rate, and enzyme activity on four occasions (days 8, 39, 69, and 100).

**Table 2:** Design of the gradual acclimation program for the long-term toxicity test.

	Control			pHS			CAS			CA-pHS		
Acclimation time (days)	Setting salinity (ppt)	Setting *C*_A_ (mmol⋅L^-1^)	Setting pH	Setting salinity (ppt)	Setting *C*_A_ (mmol⋅L^-1^)	Setting pH	Setting salinity (ppt)	Setting *C*_A_ (mmol⋅L^-1^)	Setting pH	Setting salinity (ppt)	Setting *C*_A_ (mmol⋅L^-1^)	Setting pH
D1	6	0	8.2	6	0	8.5	6	3.5	8.2	6	0	8.2
D2	6	0	8.2	6	0	8.8	6	4.5	8.2	6	1.0	8.4
D3	6	0	8.2	6	0	9.0	6	5.5	8.2	6	2.0	8.6
D4	6	0	8.2	6	0	9.2	6	6.0	8.2	6	3.0	8.8
D5	6	0	8.2	6	0	9.4	6	6.5	8.2	6	3.5	8.9
D6	6	0	8.2	6	0	9.6	6	7.0	8.2	6	4.0	9.0
D7	6	0	8.2	6	0	9.8	6	7.5	8.2	6	4.5	9.1
D8	6	0	8.2	6	0	10.0	6	8.0	8.2	6	5.0	9.2

### Analytical Methods

#### SR, PMSR, WG, SGR, and Lethal Concentration 50% (LC_50_)

PMSR, WG, and SGR were calculated as follows:

$$\begin{array}{c} \mathrm{SR}\;\left(\%\right)\;=\;100{\;}^{*}N_{e}/N_{s}\\ \mathrm{PMSR}\;\left(\%\right)\;=\;100{\;}^{*}N_{n}/N_{\left(n-1\right)}\\ \mathrm{WG}\;(\%{\mathrm{day}}^{-1})\;=\;100{\;}^{*}\;(W_{n}-W_{\left(n-1\right)})/T\\ \mathrm{SGR}\;(\%{\mathrm{day}}^{-1})\;=\;100{\;}^{*}\;(L_{n}-L_{\left(n-1\right)})/T \end{array}$$

where *N_e_* is the number of JSCs that survived in the end of 48 h acute toxicity test, *N_s_* is the number of JSCs that survived in the start of 48 h acute toxicity test, *N_n_* is the number of JSCs that survived in the *n*-th month, *N*_(_*_n_*_-1)_ is the number of JSCs that survived in the (*n*-1)-th month, *W_n_* and *W*_(_*_n_*_-1)_ are the average wet body weights of the JSCs in the *n*-th month and the (*n*-1)-th month, respectively, *L_n_* and *L*_(_*_n_*_-1)_ are the average lengths of the JSCs in the n-th month and the (*n*-1)-th month, and *T* is the duration of the experiment (months).

The value of LC_50_ for a substance is the dose required to kill half the members of a tested population after a specified test duration. LC_50_ was calculated by the linear interpolation method for acute toxicity testing (Stephan, 1977; Yao et al., 2010).

#### Oxygen Consumption Rate and Ammonia Excretory Rate

The oxygen consumption rate and ammonia excretory rate (Fan et al., 2002) were measured for the JSCs in a sealed conical flask. A dissolved oxygen analyzer (WTW Multi 3420 Set G, Xylem Inc., Germany) was used to determine the dissolved oxygen in the test water. Twenty JSCs in each group were randomly selected to determine the oxygen consumption rate and ammonia excretory rate, which were calculated as follows:

Oxygen consumption rate

$$(\mathrm{mg}\;O_{2}\;g^{-1}L^{-1}\;h^{-1})\;=\;(A_{0}-A_{1}){\;}^{*}V/\left(W^{*}T\right)$$

Ammonia excretory rate

$$(\mathrm{mg}\;g^{-1}\;h^{-1})\;=\;E/\left(W^{*}T\right),$$

where *A*_1_ and *A*_0_ are the final and initial dissolved oxygen concentrations in the test water, respectively, *V* is the volume of test water, *W* is the average wet body weight of the JSCs, *T* is the duration of the test, and *E* the ammonia nitrogen content of the test water (mg). For better labeling of results, the unit is converted to (μg g^-1^ h^-1^) in the figure after calculation.

#### Enzyme activities

The shells were removed from the JSCs and they were homogenized, before placing in an ice bath to measure the activities of enzymes comprising Na^+^/K^+^-ATPase (NKA), acetylcholinesterase (AChE), aspartate aminotransferase (AST), superoxide dismutase (SOD), and lysozyme (LZM). The homogenized tissue samples were diluted with normal saline at a ratio of 1:9 for the tissue weight (g) relative to normal saline (mL). The total protein contents of the tissue samples were determined using a Coomassie Brilliant Blue Total Protein Assay kit (Nanjing Jiancheng Bioengineering Institute, China) based on a previously reported assay (Sedmak and Grossberg, 1977).

The NKA, AChE, AST, SOD, and LZM activities were determined using an Ultra Trace Na^+^/K^+^-ATPase Assay kit, Acetylcholine Assay kit, Aspartate Amino transferase Assay kit, Superoxide Dismutase Assay kit, and Lysozyme Assay kit (Nanjing Jiancheng Bioengineering Institute, China), respectively, according to the manufacturers’ instructions. In the NKA assay, the enzyme activity unit (U) was expressed as μmol Pi mg prot^-1^ h^-1^. In the AChE assay the enzyme activity unit (U) was expressed on the basis of each mg of tissue protein incubated at 37°C for 6 min, with the hydrolysis reaction system 1 μmol matrix as a viable unit. In the AST assay, the enzyme activity unit (U) is expressed as μmol mg prot^-1^ min^-1^. In the SOD assay the enzyme activity unit (U) was expressed as the amount of enzyme corresponding to a 50% SOD inhibition rate in the reaction system.

#### Integrated Biomarker Response (IBR) Index Calculation

The activities of five enzymes (NKA, AChE, AST, SOD, and LZM) were used to calculate the IBR index, and the IBR data were expressed as a star plot area. For each month of a given test, the data processing method was as follows. For each marker, the result of the assay in each test group was calculated (*x*_ii_) to determine the total group average (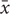) and total group standard deviation (*s*). Next, the value of *x*_i_ for each group was normalized as follows: x_i_′ = (x_i_ -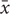)/s, where, *x*_i_′ are normalized data for *x*_i_. If the activity of a marker is activated by stress, let *Z* = *x*_i_′; otherwise, let *Z* = -*x*_i_′. Then, let |*x*_min_| = the absolute value of the minimum of the marker homogeneity data in all groups. The score for each biomarker in each group was determined as: *B_i_* = Z+|*x*_min_|. The star plot figure was prepared where the *B_i_* value for each marker in a group was the length of the radiation. The IBR values for each group were obtained by calculating the area of the star plot (the sum of the areas *A_i_* that the triangles surrounded due to the radiation of adjacent biomarkers in the figure):

$$\begin{array}{c} \mathrm{IBR}\;=\;\overset{n}{\underset{i=1}{\sum }}A_{i}(Beliaeffand Burgeot, 2002)\;\\ A_{i}\;=\;(S_{i}^{*}S_{i+1}^{*}\;\mathrm{Sin}\alpha )/2,\;(Devin et al., 2014)\\ \;\alpha \;=\;2\pi /K \end{array}$$

Where *K* is the number of biomarkers used in the experiment.

#### Statistical Analysis

Statistical analyses were performed using SPSS 19.0 statistical software. All data were subjected to Tukey’s test of one-way ANOVA which determines significant differences among treatments. Figures were plotted using Sigmaplot 12.3.

## Results

### Acute Toxicity Test

#### Acute Toxicity Test With Different pH Values

**FIGURE 1:**
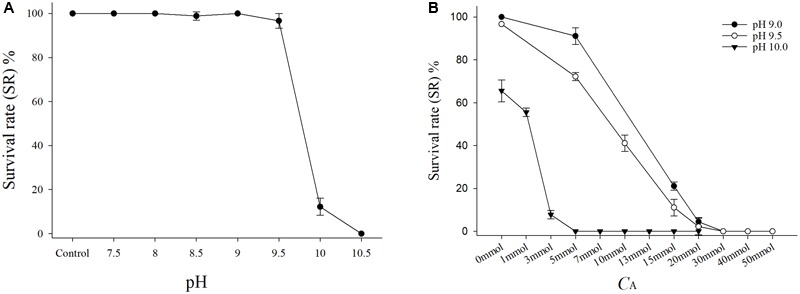
**(A)** Survival rate of juvenile of *Sinonovacula constricta* (JSCs) with different pH values under the same *C*_A_ in the acute toxicity test. Bars represent the mean ± SE (*n* = 3). The control group was normal artificial seawater (ASW) with no adjustments to the pH and *C*_A_. **(B)** Survival rates of JSCs with different *C*_A_ levels under the same pH in the acute toxicity test. Bars represent the mean ± SE (*n* = 3).

After the stress test for 48 h (**Figure 1A**), the SRs of the JSCs at pH values ranging from 7.5 to 9.0 were nearly 100% at 2.5 mmol L^-1^ *C*_A_, and there were no significant differences compared with the control group. The SRs of the JSCs at pH 9.5 were higher than 90%, but they decreased greatly at pH 9.5–10.0. All of the JSCs died within 24 h when the pH reached 10.5. The 48 h LC_50_ for this test was pH 9.86 (**Table 3**).

**Table 3:** The 48 h LC_50_ results for Juvenile of *Sinonovacula constricta* (JSCs) in the acute toxicity test.

Different pH under the same *C_A_*		Different *C_A_* under the same pH	
*C_A_* (mmol⋅L^-1^)	LC_50_ of pH	pH	LC_50_ of *C_A_* (mmol⋅L^-1^)
2.5	9.86	9.0	10.38
–	–	9.5	8.79
–	–	10.0	3.11

#### Acute Toxicity Test of JSCs With Different *C*_A_ Values at the Same pH

Under the same *C*_A_ conditions, after the stress test for 48 h (**Figure 1B**), the SRs of the JSC decreased in the groups as the pH increased. In addition, the SRs of the JSCs decreased in the groups as the *C*_A_ increased under the same pH conditions. In the groups with pH 9.0 and 9.5, the SRs of the JSCs were 0% when *C*_A_ exceeded the set value of 20 mmol L^-1^. Moreover, the SR of JSCs was 0% when the pH was 10.0 and *C*_A_ exceeded the set value of 3 mmol L^-1^. The 48 h LC_50_ values with pH 9.0, pH 9.5, and pH 10.0 were 10.38, 8.79, and 3.11 mmol L^-1^, respectively (**Table 3**).

### Long-Term Toxicity Test

#### Long-Term Toxicity Test Based on the PMSR of JSCs

**FIGURE 2:**
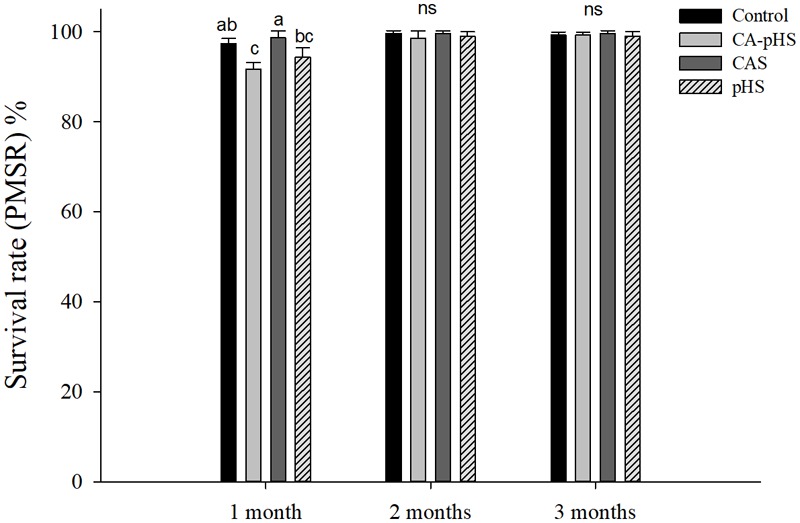
Juvenile of *S. constricta* per month survival rate in the long-term toxicity test. Bars (mean ± SE, *n* = 3) with different letters indicate significant differences (*p* < 0.05) among the four groups in each month. The letters “ns” indicate insignificant difference among the four groups in each month.

During the 100-day long-term stress test, the surviving numbers of JSCs were counted every month (**Table 4**). The ultimate absolute average survival rates for JSCs after 100 days in each group were 96.67, 89.67, 98.00, and 92.33% for the control, CA-pHS, CAS, and pHS groups, respectively. Survival rate was significantly lower in the CA-pHS group than the control group (*p* < 0.05), but the survival rates in the other two groups did not differ significantly compared with the control group. According to the PMSR data for each month (**Figure 2**), the PMSR in the first month differed among groups. The PMSR in the CA-pHS group was significantly lower than that in the control group (*p* < 0.05). The PMSRs in the other two groups did not differ significantly compared with that in the control group. Four test groups maintained high PMSRs from the second month onwards and there were no significant differences between them.

**Table 4:** Growth and death of JSCs in different treatment groups in the long-term toxicity test.

Index	Groups	Rearing period		
		1 month	2 months	3 months
Shell length (cm)	Control	0.938 ± 0.115^a^	1.029 ± 0.120^a^	1.221 ± 0.075^a^
	CA-pHS	0.819 ± 0.135^c^	0.885 ± 0.061^c^	1.170 ± 0.083^c^
	CAS	1.021 ± 0.134^b^	1.130 ± 0.126^b^	1.296 ± 0.112^b^
	pHS	0.972 ± 0.187^ab^	1.060 ± 0.108^a^	1.282 ± 0.095^b^
Body weight (g)	Control	0.057 ± 0.003^a^	0.082 ± 0.002^a^	0.121 ± 0.002^a^
	CA-pHS	0.034 ± 0.003^b^	0.060 ± 0.001^b^	0.099 ± 0.006^b^
	CAS	0.061 ± 0.001^a^	0.086 ± 0.002^a^	0.131 ± 0.003^a^
	pHS	0.060 ± 0.003^a^	0.083 ± 0.005^a^	0.127 ± 0.004^a^
The number of deaths	Control	2.667 ± 1.155^ac^	0.333 ± 0.577^a^	0.667 ± 0.577^a^
	CA-pHS	8.333 ± 1.528^b^	1.333 ± 1.528^a^	0.667 ± 0.577^a^
	CAS	1.333 ± 1.528^c^	0.333 ± 0.577^a^	0.333 ± 0.577^a^
	pHS	5.667 ± 2.082^ab^	1.000 ± 1.000^a^	1.000 ± 1.000^a^

*Values (mean ± SE) with different letters denote significant differences (p < 0.05) among the four groups for the same index in each month*.

#### Long-Term Toxicity Test Based on JSC Growth

**FIGURE 3:**
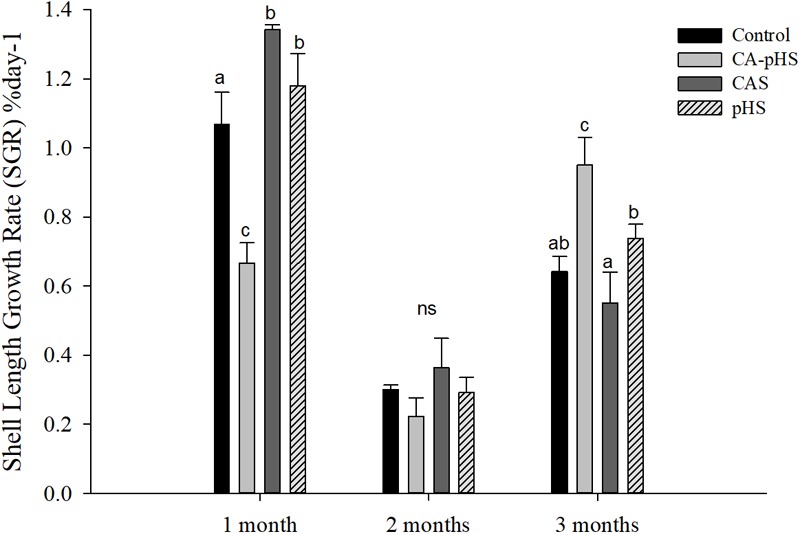
Juvenile of *S. constricta* shell length growth rate (SGR) in the long-term toxicity test. Bars (mean ± SE, *n* = 3) with different letters denote significant differences (*p* < 0.05) among the four groups in each month. The letters “ns” indicate insignificant difference among the four groups in each month.

**FIGURE 4:**
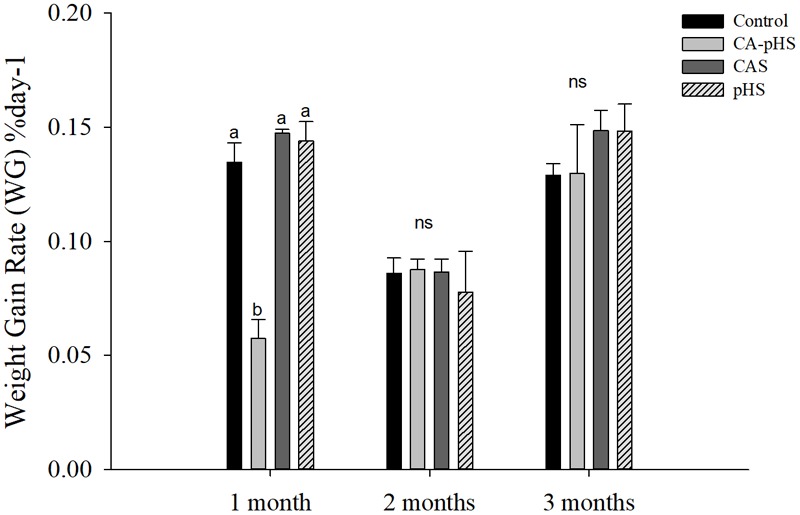
juvenile of *S. constricta* body weight gain rate in the long-term toxicity test. Bars (mean ± SE, *n* = 3) with different letters denote significant differences (*p* < 0.05) among the four groups in each month. The letters “ns” indicate insignificant difference among the four groups in each month.

**Table 4** shows the direct measurements of the body weight and shell length each month. In the long term, the growth rate was calculated based on the increase in the body weight and shell length by JSCs each month. The body weight and shell length in the CA-pHS group were significantly lower than the control group after 100 days stress rearing. The shell length was significantly higher in the CAS group than the control group. Details of the growth rates are shown in **Figures 3, 4** as %day^-1^ for SGR and WG. **Figure 3** shows that the SGR in the pHS group did not differ significantly from that in the control group after 3 months, but it was higher than that in the control group during the first month and the third month. The SGR was significantly higher in the CAS group than the control group during the first month, but it did not differ significantly from that in the control group during the second month and the third month. The SGR was significantly lower in the CA-pHS group than the control group during the first month, but it was significantly higher than that in the control group during the third month. **Figure 4** shows that the WG was significantly slower in the CA-pHS group than the control group during the first month, but they did not differ significantly during the second and third months. The WG in the control group did not differ significantly from those in the CAS and pHS groups during all months.

#### Long-Term Toxicity Tests Based on Oxygen Consumption and Ammonia Excretory Rate

**FIGURE 5:**
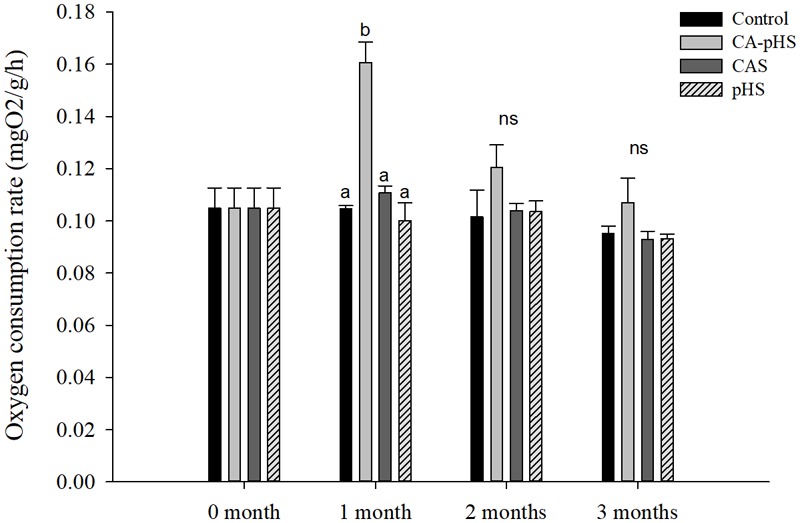
juvenile of *S. constricta* oxygen consumption rate in the long-term toxicity test. Bars (mean ± SE, *n* = 3) with different letters denote significant differences (*p* < 0.05) among the four groups in each month. The letters “ns” indicate insignificant difference among the four groups in each month.

**FIGURE 6:**
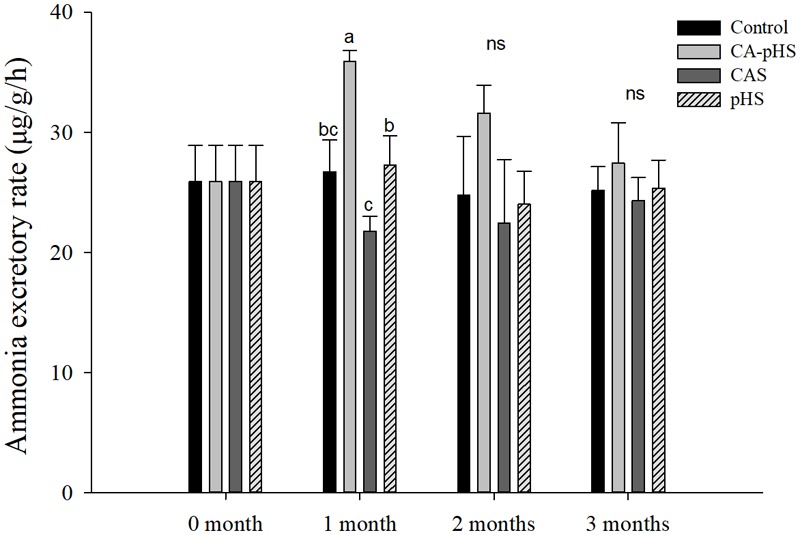
Juvenile of *S. constricta* ammonia excretory rate in the long-term toxicity test. Bars (mean ± SE, *n* = 3) with different letters denote significant differences (*p* < 0.05) among the four groups in each month. The letters “ns” indicate insignificant difference among the four groups in each month.

**Figure 5** shows that there were no significant differences in the oxygen consumption rate among the groups, except for the CA-pHS group in the first month when it was significantly higher than that in the control group. The oxygen consumption rate was higher in the CA-pHS group than that in the control group during the second and third months but the difference was not significant. In **Figure 6**, the results are similar to those in **Figure 5**, except that the ammonia excretory rate in the CAS group during the first month was significantly lower than that in the pHS group and the CA-pHS group. The oxygen consumption rate (**Figure 5**) and ammonia excretory rate (**Figure 6**) decreased with time in the CA-pHS group.

#### Long-Term Toxicity Test Based on Enzyme Activities

**FIGURE 7:**
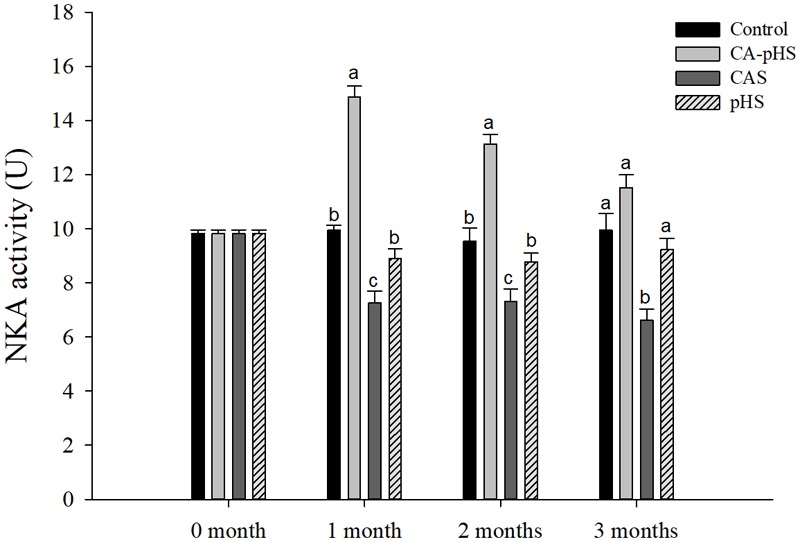
Juvenile of *S. constricta* Na^+^/K^+^-ATPase (NKA) activity levels in the long-term toxicity test. Bars (mean ± SE, *n* = 3) with different letters denote significant differences (*p* < 0.05) among the four groups in each month. The letters “ns” indicate insignificant difference among the four groups in each month.

**Figure 7** shows that the NKA activity in the first month differed significantly among the groups. Compared with the control group, the NKA activity was significantly higher in the CA-pHS group, but significantly lower in the CAS and pHS groups. The NKA activity decreased with time in the CA-pHS group, but it did not differ significantly compared with the control group in the third month. The NKA activity was still significantly lower in the CAS group compared with the control group in the second and third month, and it tended to decline with time. In the second and third month, there was no significant difference in the NKA activity between the pHS group and the control group.

**FIGURE 8:**
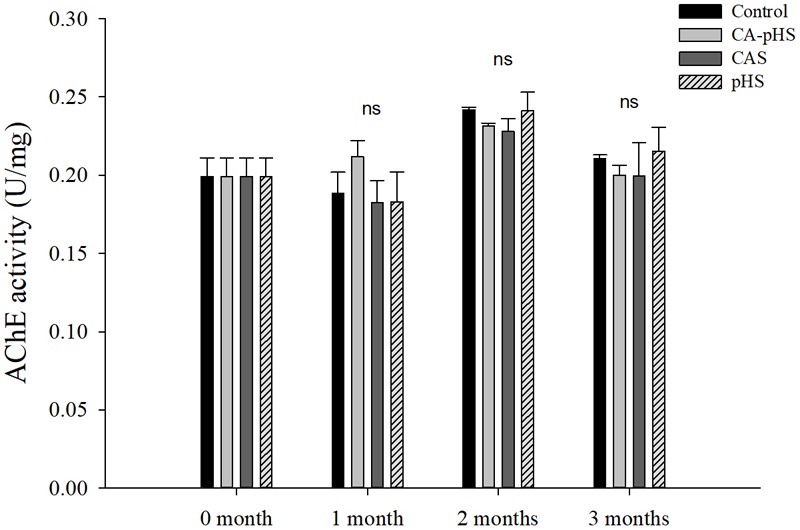
Juvenile of *S. constricta* acetylcholinesterase (AChE) activity levels in the long-term toxicity test. Bars (mean ± SE, *n* = 3) with different letters denote significant differences (*p* < 0.05) among the four groups in each month. The letters “ns” indicate insignificant difference among the four groups in each month.

**FIGURE 9:**
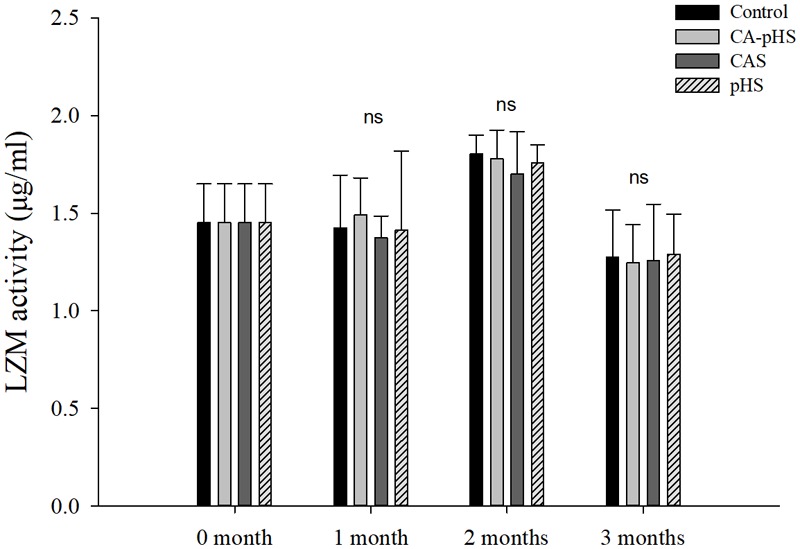
Juvenile of *S. constricta* lysozyme (LZM) activity levels in the long-term toxicity test. Bars (mean ± SE, *n* = 3) with different letters denote significant differences (*p* < 0.05) among the four groups in each month. The letters “ns” indicate insignificant difference among the four groups in each month.

**Figures 8, 9** shows that there were no significant differences in the AChE activitiy and LZM activity among groups in each month.

**FIGURE 10:**
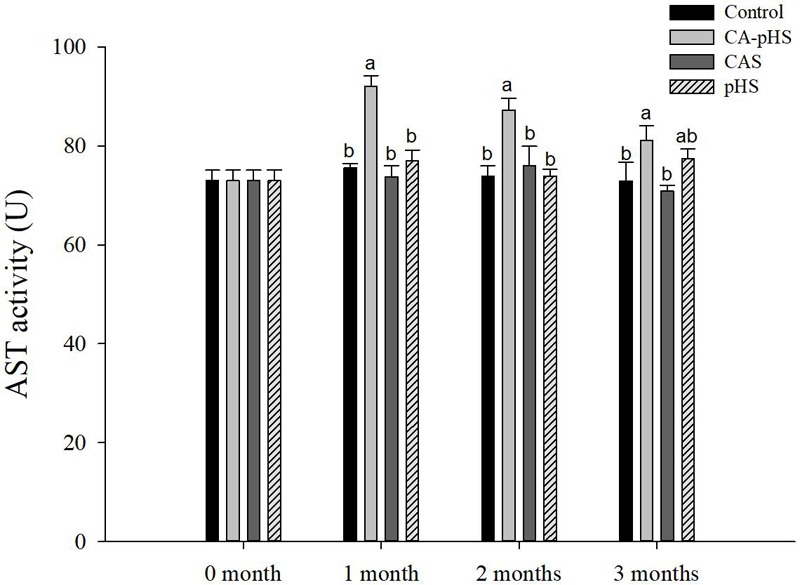
Juvenile of *S. constricta* aspartate aminotransferase (AST) activity levels in the long-term toxicity test. Bars (mean ± SE, *n* = 3) with different letters denote significant differences (*p* < 0.05) among the four groups in each month. The letters “ns” indicate insignificant difference among the four groups in each month.

**Figure 10** shows that the AST activity was significantly higher in the CA-pHS group than the control group in each month. The AST activity in the CA-pHS group tended to decline with time, and there was no significant difference between the AST activity in the CA-pHS group and the pHS group during the third month.

**FIGURE 11:**
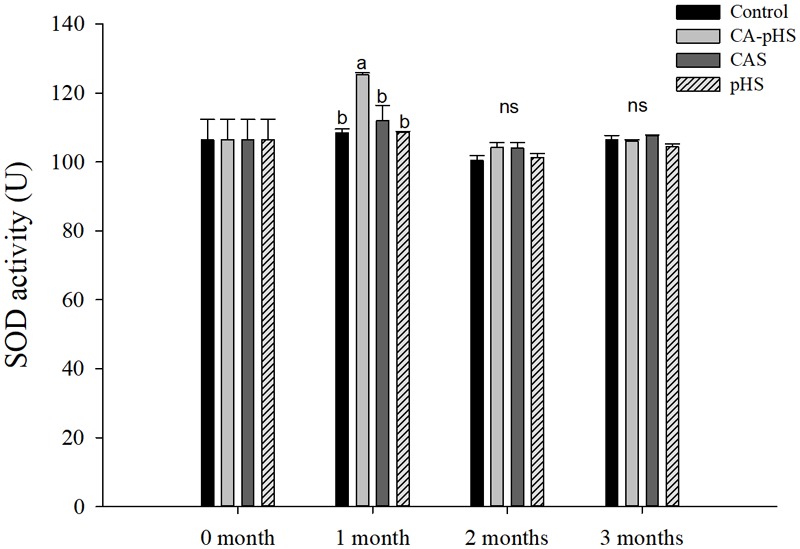
Juvenile of *S. constricta* superoxide dismutase (SOD) activity levels in the long-term toxicity test. Bars (mean ± SE, *n* = 3) with different letters denote significant differences (*p* < 0.05) among the four groups in each month. The letters “ns” indicate insignificant difference among the four groups in each month.

**Figure 11** shows that the SOD activity in the CA-pHS group differed significantly compared with that in the control group during the first month, but there were no significant differences among the other groups.

#### Long-Term Toxicity Test Based on the IBR Index

**FIGURE 12:**
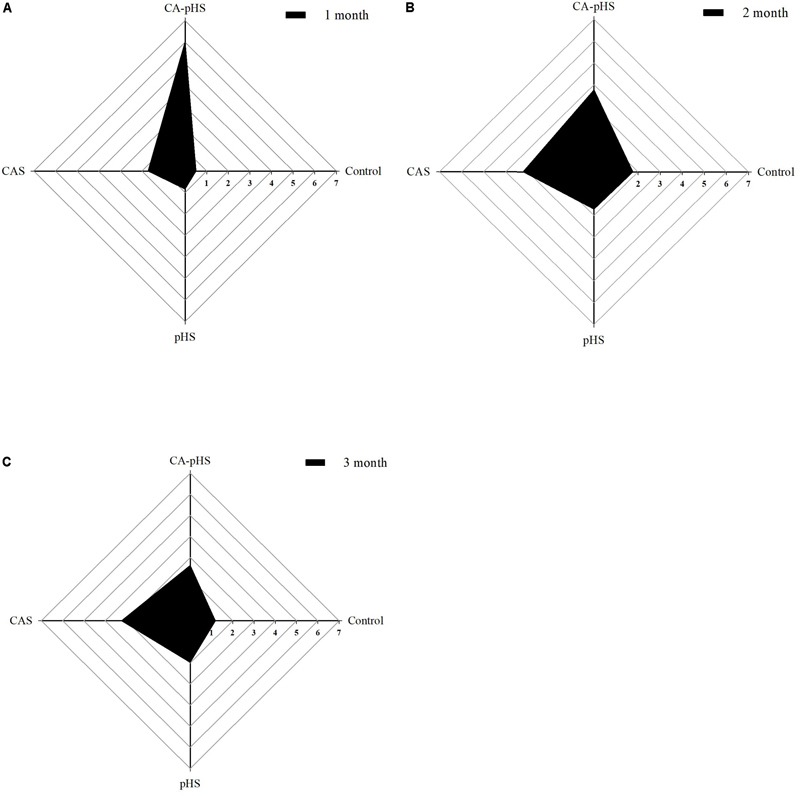
Juvenile of *S. constricta* integrated biomarker response (IBR) values in the long-term toxicity test. **(A–C)** Represent month 1, month 2, and month 3, respectively. Axis values are expressed as IBR values.

The IBR indices were calculated using the data sets obtained after 1, 2, and 3 months (**Figure 12**). The IBR value was much higher in the CA-pHS group than the other groups during the first month, and the IBR values in the four groups could be ranked as: CA-pHS > CAS > pHS > control. The IBR values were similar in the CA-pHS and the CAS groups during the second month, and similar in the pHS and control groups, where the IBR values for the four groups could be ranked as: CA-pHS > CAS > pHS ≈ control. In the third month, the IBR value was larger for the CAS group than the other groups, and the IBR values for the four groups could be ranked as: CAS > CA-pHS > pHS > control. The IBR value in the CA-pHS group exhibited a decreasing trend from the first month to the second month relative to the IBR value for the control group. The IBR values obtained for the CA-pHS group in the second month and third month varied little compared to those in the control group. The IBR values in the pHS group and CAS group were higher than those in control group at all times, except the IBR value for the pHS group during the second month was similar to that for the control group. The peak IBR value occurred in the first month in the CA-pHS group.

## Discussion

*Sinonovacula constricta* is an estuarine intertidal zone shellfish and faces many challenges, such as the water quality changes, during its life cycle. Therefore, it was selected as a potential shellfish for breeding in ISWs due to its high tolerance of a broad temperature range and different salt concentrations. Previously, Lin and Wu (1984) reported that the development of *S. constricta* larvae occurs in the metamorphosis phase between 2 and 40°C, and between salinity levels of 1.8 and 40 ppt, but the larvae could survive for several hours in water at -2°C and the survival rate was 3.2% in water at 1.8 ppt. The tolerance levels of *S. constricta* may be higher than generally considered in terms of long-term low-salt stress tolerance (unpublished data), and the source (living environment) of the parent is also an important reason for determining the level of tolerance of animals (Alden, 1964). In summary, the temperature (annual scope from -10.2–20.2°C) and salinity (average of 6 ppt) data range of the target ISW were considered that may not be an important influence factor (stress factors), in this study. Thus, we tested pH and *C*_A_ as the key factors in order to study their effects on the growth and physiology of *S. constricta*.

The key factors that limit the growth and reproduction of farmed animals in ISWs have been investigated widely. In terms of the ions involved in ISWs, CO_3_^2-^ and HCO_3_^-^ (*C*_A_), OH^-^ (pH), Na^+^, K^+^, Ca^2+^, Mg^2+^, Cl^-^, and SO_4_^2-^ can all affect the growth and development of farmed animals (Rijstenbil and Gerringa, 2002; Partridge, 2008; Dinh, 2015). Hiele et al. (2014) suggested that imbalanced ion ratios in ISWs can have major effects on the growth of mussels. Fang et al. (2000) and Yang et al. (2004) reported that high *C*_A_ and high pH levels are major factors that can restrict aquaculture in ISWs. Studies have shown that the extreme pH value is in the range of 7.08–9.67 and the extreme *C*_A_ value is in the range of 1.73–12.02 mmol L^-1^, in northwestern China (Shi, 2009). The results of the acute test showed that the SRs of JSCs under the same *C*_A_ conditions were not affected by pH in the range of 7.0–9.5. However, when the pH exceeded 9.5, the SR of the JSCs was low after 48 h. Under the same pH, the SRs of the JSCs decreased significantly as the *C*_A_ concentration increased, where the pH and *C*_A_ had synergistic effects. According to Dennis (1974) and Wen (2009), the body fluids of aquatic animals tend to maintain a near-neutral pH because cells and organisms are able to tolerate a range of changes in the external pH due to their *in vivo* buffer system. Thus, when the pH changes in the external environment, the chemical buffer system can maintain the balance. However, when it exceeds the buffer limit, the pH value of the body fluids will change drastically and affect the normal physiological activities of the organism. Zheng et al. (2005) found that elevated water *C*_A_ can cause surface damage to the gill tissues of aquatic animals as well as affecting the function of the outer surface of the gill epidermal cells, which exchange Cl^-^ and HCO_3_^-^. Galat et al. (2010) also found that exposure to a high level of *C*_A_ for a short time could lead to the proliferation or hypertrophic gill chloride cells. Ye et al. (2011) showed that the survival and growth of *Mytilus coruscus* larvae are influenced by pH, where the survival and growth of the larvae were significantly inhibited when the pH was 10.0. This result may be attributed to the influence of the environmental pH on the blood pH in the body. Therefore, a pH range of 9.5–10.0 may be the maximum range tolerated by the *in vivo* buffer system of JSCs, where acute death occurs when the pH exceeds 10.5 because the buffer system is completely disrupted (Wen, 2009). Acute stress tests have shown that exposure to high *C*_A_ concentrations may damage the gill tissue within a short period of time, thereby resulting in acute death of JSCs (Alper et al., 2002). However, JSCs are also resistant to certain concentrations of *C*_A_ for a short period of time as well as the pH. There is a balance between pH and *C*_A_ in water: OH^-^ + HCO_3_^-^→ CO_3_^2-^ + H_2_O, where both elevated pH or elevated *C*_A_, will lead to elevated CO_3_^2-^, and CO_3_^2-^ has a high toxicogenic effect on aquatic animals (Lei et al., 1985; Yao et al., 2010; Zhao et al., 2014). Therefore, according the results of the acute tests, pH and *C*_A_ have a synergistic lethal effect (Wilkie and Wood, 1996).

We designed the long-term toxicity test according to the results of the acute test. The long-term toxicity test comprised slow acclimation to the stress conditions by all groups during the first 8 days (**Table 2**), followed by continuous stress for 3 months. According to the PMSR data (**Table 4** and **Figure 2**), highly toxic death and growth inhibition occurred in the first month, followed by a stable mortality rate and few deaths in the different groups. Thus, the combined lethal effect of pH and *C*_A_ had the greatest influence, followed by pH, but *C*_A_ did not affect the survival rate. The same trend was also reflected in the SGR and WG results, but unlike the mortality results, the pH and *C*_A_ did not affect the growth rate. McGraw et al. (2002) and Jayasankar et al. (2009) suggested that a period of acclimation can promote the balance between the physiological environment and the surrounding environment for aquatic animals, thereby reducing the osmoregulatory stress in animals and increasing survival. Zhao et al. (2016) studied the effects of *C*_A_ on osmolality in Nile tilapia and showed that the effect of *C*_A_ on the osmotic pressure was acute, with the maximum impact occurring in 24 h before decreasing. In a study of the effects of pH on the survival of *Moerella iridescens* juveniles, Gu et al. (1998) found that there was a dramatic change in survival after 5 days (45% on day 5) at pH 10.0, but the survival rate was basically stable from days 5–14 (40% on days 10 and 14), thereby indicating their strong adaptation to a high pH. These observations are consistent with the results of the acute and chronic stress tests conducted in the present study. According to Portz et al. (2006), the pH can directly affect the composition of HCO_3_^-^ and CO_3_^2-^ in aquatic animals to influence physiological metabolism, but this effect can be reduced under long-term stress (Portz et al., 2006; Parra and Baldisserotto, 2007). In the present study, the pH had a greater effect on the survival of JSCs but it did not influence growth, whereas only increasing the *C*_A_ did not affect survival and growth in the JSCs. Under a high pH, a large amount of CO_3_^2-^ was present in the CA-pHS group, where it had significant adverse effects on the survival and growth of JSCs. However, most of the JSCs could tolerate these stresses in the long-term test and they adapted to the changed environments (high pH and *C*_A_), where the finally survival rates of the JSC exceeded 85% in each group.

Continuous monitoring of physiological indicators in each of the long-term test groups indicated the physiological effects of pH and *C*_A_ on JSCs. Excluding AChE and LZM (which were not significantly affected by pH and *C*_A_), the other indices differed significantly during the first month but the differences decreased in the second and third months. In the first month, the combined effect of pH and *C*_A_ caused significant increases in the oxygen consumption rate and ammonia excretory rate. Thomas and Poupin (1985) found that high levels of *C*_A_ induced mixed respiratory and metabolic alkalosis in adult rainbow trout. Pan and Jiang (2002) found a similar effect in shrimp where a high level of CO_3_^2-^ production hindered the gill surface Cl^-^/HCO_3_^-^ and Na^+^/H^+^ exchange, and the metabolism accelerated and oxygen consumption increased in order to maintain the internal balance. Zhao et al. (2016) showed that changes in the pH and *C*_A_ upregulated ammonia nitrogen metabolism-related genes in Nile tilapia. These results agree with our AST activity test results, as shown in **Figure 10**. Fan et al. (2002) studied the acute effects of pH on *S. constricta* and found that a high pH had no significant effect on the oxygen consumption rate but it had a significant effect on the ammonia excretion rate. By contrast, we found no significant change in the ammonia excretion rate in the pHS group (**Figure 6**), possibly due to the rapid recovery of ammonia nitrogen excretion after acclimation for 1 month. Changes in pH can damage osmotic regulation in mollusks and even cause disease outbreaks, with a large number of deaths and other phenomena (Breitwieser et al., 1987; Zhang et al., 2008). Pan et al. (2004) studied the effects of pH on the activity of NKA in the gill filaments of *L. vannamei*. When the pH varied over 3 days, the NKA activity in the gill filaments of *L. vannamei* reached the maximum value at 12 h under high pH, but it returned to normal after 3 days. **Figure 7** shows that the NKA activity could adjust rapidly in a short time when only the pH increased, which caused osmotic changes *in vivo*. An increase in HCO_3_^-^ ions may continue to inhibit the activity of NKA. However, an increase in CO_3_^2-^ ions may upregulate the activity of NKA over a longer period of time. Wang et al. (2013) found that under high *C*_A_ stress, *Gymnocypris przewalskii* could respond to changes in the external environment by regulating the activities of SOD, alkaline phosphatase, and acid phosphatase in the liver and kidney. In addition, studies have shown that as the pH increases, the bacteriolytic activity of shrimp decreases and the activity of phenoloxidase increases (Ha et al., 2009). We also found that the SOD activities increased in the JSCs, but pH or *C*_A_ did not affect the LZM activity in the long term. The conduction and *in vivo* responses to stress signals in fish are considered to function via the endocrine system and through the nervous system (Portz et al., 2006). The nervous system of fish is more developed than that of shellfish, but it still plays a major role in shellfish physiology (Croll and Dickinson, 2004; Dewilde et al., 2006). The nervous system involves a cascade of reactions (Wayne, 2001) and the results shown in **Figure 8** suggest that AChE may act as an upstream regulator of the activities of other enzymes and physiological responses, although the AChE activity was only higher in the CA-pHS group compared with the other groups (but not significantly different) during the first month.

The IBR index was established by Beliaeff and Burgeot (2002). The IBR values can be obtained for different groups by calculating the area of the star plot in order to distinguish the effects of the degree of pollution among groups (Bodin et al., 2004; Oliveira et al., 2010). Jiang et al. (2015) described the effects of the virulence of No. 0 fuel on *S. constricta* using the IBR index. In contrast to normal seawater, the target ISW features (high pH and *C*_A_) could be considered as environmental stressors (Portz et al., 2006). In the present study, we used the IBR index to determine the combined effects of pH and *C*_A_ in water. **Figure 12** shows that the trends in the IBR values during the first month and second month were similar but they could be ranked as: CA-pHS group > CAS group > pHS group > control group. However, compared with the second month, the IBR value for the CA-pHS group was higher than that for the CAS group during the first month. The areas enclosed by the IBR values for each group in the star plot decreased in size from the first month to the third month, thereby indicating that the tolerance of the JSCs to high pH and *C*_A_ increased under the long-term stress.

Obviously, this series of physiological changes are interrelated, high level CO_3_^2-^ and OH^-^ were present in the CA-pHS group first hinders the gill surface Cl^-^/HCO_3_^-^ and Na^+^/H^+^ exchange and causes more complete (Compared to groups CAS and pHS) osmotic pressure imbalance. Therefore, resulting in increased NKA activity. The increase in NKA activity leads to a rise of metabolic consumption and then leads to increase in oxygen consumption, ammonia excretion, AST activity, and metabolic free radicals, further leading to SOD activity increase. The JSC individuals who can tolerate the combined effects of these factors have survived, but at the expense of part of the growth energy. However, such effects will be adapted by some JSC in the long-term stress.

## Conclusion

In this study, the data showed consistently that high *C*_A_ and pH levels could induce physiological changes in the JSC within a short time, and even cause death or slow growth, where there was a synergistic effect between *C*_A_ and pH. However, the JSCs adapted well to these changes under long-term stress. Thus, *S. constricta* may be a suitable species for breeding in ISWs in China. Other important factors (Na^+^, K^+^, Ca^2+^, Mg^2+^) in the ISW that influence the survival, growth, and physiology of the *S. constricta* will be the goals of our next study. We intend to unravel the molecular mechanisms of *S. constricta* adaptation to the environment of ISW through more in-depth research. In addition, we are very interested in whether *S. constricta* can affect the content of some ions (such as Ca^2+^ and Mg^2+^) in ISW by exerting the function of bivalve (biomineralization), so to explore the possibility of *S. constricta* as an environmental regulator for ISW. Further practice, cultivation of *S. constricta* in ISW in Northwest China, is imminent and significant.

## Author Contributions

PM was responsible for experimental design, test operations, data processing, and article writing. YB was responsible for test operations and data processing. LX was responsible for experimental design and article modification. LT was responsible for experimental data collection. LJ, ND, and DZ were responsible for the experimental program guidance.

## Conflict of Interest Statement

The authors declare that the research was conducted in the absence of any commercial or financial relationships that could be construed as a potential conflict of interest.
